# Investigation of acyclic uridine amide and 5′-amido nucleoside analogues as potential inhibitors of the *Plasmodium falciparum* dUTPase^☆^

**DOI:** 10.1016/j.bmc.2013.07.004

**Published:** 2013-09-15

**Authors:** Shahienaz E. Hampton, Alessandro Schipani, Cristina Bosch-Navarrete, Eliseo Recio, Marcel Kaiser, Pia Kahnberg, Dolores González-Pacanowska, Nils Gunnar Johansson, Ian H. Gilbert

**Affiliations:** a Division of Biological Chemistry and Drug Discovery, College of Life Science, University of Dundee, Sir James Black Centre, Dundee DD1 5EH, UK; b Instituto de Parasitología y Biomedicina, Consejo Superior de Investigaciones Científicas, Parque Tecnológico de Ciencias de la Salud, Avenida del Conocimiento, 18016 Armilla, Granada, Spain; c Swiss Tropical and Public Health Institute, Socinstrasse 57, CH-4002 Basel, Switzerland; d University of Basel, Petersplatz 1, CH-4051 Basel, Switzerland; e Medivir AB, P.O. Box 1086, S-14122 Huddinge, Sweden

## Abstract

Previously we have shown that trityl and diphenyl deoxyuridine derivatives and their acyclic analogues can inhibit *Plasmodium falciparum* dUTPase (*Pf*dUTPase). We report the synthesis of conformationally restrained amide derivatives as inhibitors *Pf*dUTPase, including both acyclic and cyclic examples. Activity was dependent on the orientation and location of the amide constraining group. In the case of the acyclic series, we were able to obtain amide-constrained analogues which showed similar or greater potency than the unconstrained analogues. Unfortunately these compounds showed lower selectivity in cellular assays.

## Graphical abstract

**Figure:**
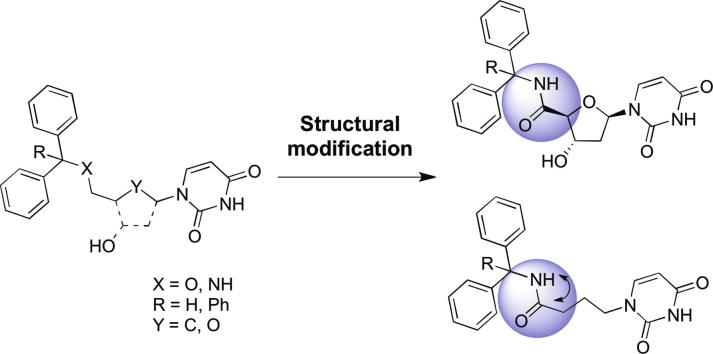

## 1 Introduction

Malaria is a major global problem, affecting millions of people each year. If not treated promptly; malaria can kill rapidly, especially children. In 2010, this accounted for an estimated 655,000 global malaria deaths (possibly ranging up to almost 1 million), 91% of which were in the African region.^1,2^ Following the announcement of the goal of the global elimination of malaria; there is an urgent need for novel drug targets in order to overcome the current problems of resistance in the available antimalarial drugs^3,4^ and to address new aspects of the disease.

Deoxyuridine 5′-triphosphate nucleotidohydrolase (dUTPase) is the enzyme that catalyses the hydrolysis of dUTP to dUMP. This provides dUMP, a precursor required for the biosynthesis of dTTP, whilst controlling the dUTP:dTTP concentration within the cell to levels that will prevent mis-incorporation of dUTP into DNA.^5^ Due to the essentiality of dUTPases for cell viability in all organisms studied to date, including *Escherichia coli* and *Saccharomyces cerevisiae*,^6–8^ it is therefore likely that dUTPases represent a novel target that yet remains to be explored in malaria.

We have previously reported the synthesis and biological evaluation of tritylated acyclic uracil analogues,^9–11^ which were shown to be potent and selective inhibitors of the *Plasmodium falciparum* dUTPase (*Pf*dUTPase). Interestingly these analogues, which were synthesised with varying chain lengths (Table 1), showed comparable activities to their preceding cyclic counterparts,^12^ therefore are of equal interest in terms of inhibitory activities. More recently in an attempt to decrease lipophilicity and increase water solubility, we have shown when replacing the trityl group with a diphenyl moiety, in both acyclic and cyclic molecules, that it is possible under certain circumstances to retain *Pf*dUTPase enzyme inhibition^13^ (Table 1 and Table 2).

A clear advantage of the acyclic analogues is that they have reduced molecular weight in addition to decreased *c *log* P* values, therefore are possibly better candidates for the synthesis of an oral compound.^14^ Additionally, these derivatives lack rigidity in their structure, thus could allow access to binding pockets not accessible with more rigid templates. The downsides of this strategy are entropic disadvantages and the possibility of multiple binding modes. One way in which to overcome this problem is to try and conformationally restrain the flexible chain by the insertion of one or more functional groups that are restricted in their rotation, thereby introducing a certain degree of rigidity. Appropriate choice of functional group may also give additional interactions with the active site, which may lead to an increase in potency and possibly also selectively. Additionally there is also potential for alteration and improvement of the pharmacokinetic properties of these compounds as anti-parasitic agents.

**Figure 1:**
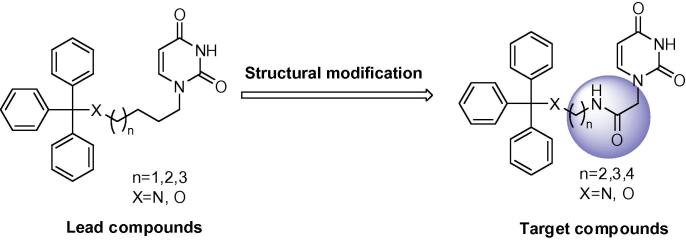
Derivatives synthesised by McCarthy et al.^10^ with the C-2,3 amide functionality.

We have previously synthesised within our laboratories mono alkyl chain uracil acetamides with the amide bond insertion into the alkyl linker chain at the C-2,3 position (Fig. 1).^10^ These were shown to exhibit weak inhibition of the *Pf*dUTPase; therefore it did not seem that an amide linkage at this position was favourable.

**Figure 2:**
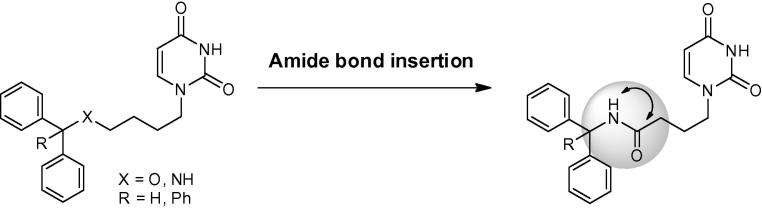
Proposed structural modification to linker chain at C-4 position.

Here we describe insertion of the amide bond at the C-4 position into the alkyl linker chain in order to probe the effects of a movement in the amide linkage in the tritylated derivatives. Diphenyl analogues were also included in this study, and only the 4C chain was synthesised as they were shown to have optimal activity and selectivity in the straight chain tritylated derivatives (Table 1). Additionally reversal of the amide linkage was also investigated (Fig. 2).

**Figure 3:**
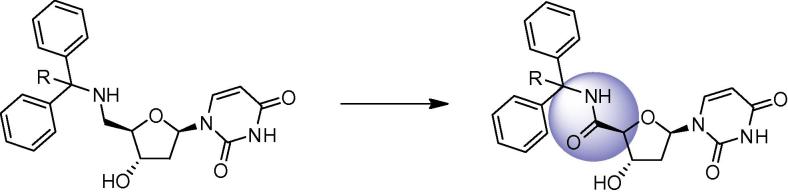
Proposed insertion of amide bond into cyclic nucleoside.

Finally insertion of the amide bond into the cyclic compounds gave an overall comparison of the effect of this increased rigidity in the restrained nucleoside upon enzyme inhibition (Fig. 3).

### 1.1 Chemistry

#### 1.1.1 Acyclic analogues

**Scheme 1:**
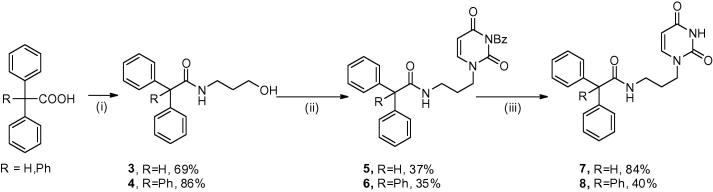
Synthesis of acyclic uridine amide analogues: (i) NH_2_(CH_2_)_3_OH, HOBt, TBTU, DIPEA, DMF, rt,16 h; (ii) 3*N*-benzoyl uracil, PS-PPh_3_, DIAD, THF, rt, 16 h; (iii) 0.2 M NaOMe, CH_3_OH, rt, 16 h.

The overall synthetic strategy (Scheme 1) in order to synthesise the first set of amides involved coupling of the relevant carboxylic acids to the amino alcohol linker chain, followed by attachment to the uracil base.

The initial amidation step was achieved starting from either diphenylacetic acid or triphenylacetic acid. HOBt and TBTU were used as coupling reagents with 3-aminopropanol to give intermediate compounds **3** and **4**. Mitsunobu coupling using polymer supported triphenylphosphine was carried out in order to attach the nucleobase, prior to deprotection of the benzoyl group yielding final compounds **7** and **8**.

**Scheme 2:**
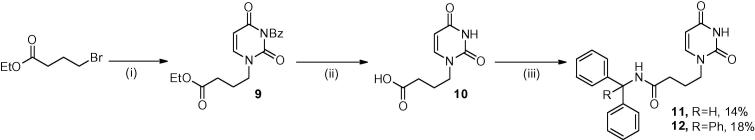
Synthetic route to acyclic derivatives with amide bond reversed: (i) 3*N*-benzoyl uracil, Cs_2_CO_3_, DMF, 60 °C, 1 h, 88%; (ii) 1 M NaOH, THF, rt, 16 h 65%; (iii) TrtNH_2_ or (C_6_H_5_)_2_(CH)NH_2_, HOBt, TBTU, DIPEA, DMF, rt,16 h.

The synthesis was modified slightly in order to produce the corresponding compounds with the amide bond reversed (Scheme 2). Following ester hydrolysis of **9**,^13^ amidations of the resultant acid gave the desired compounds, **11** and **12**.

#### 1.1.2 Cyclic analogues

**Scheme 3:**
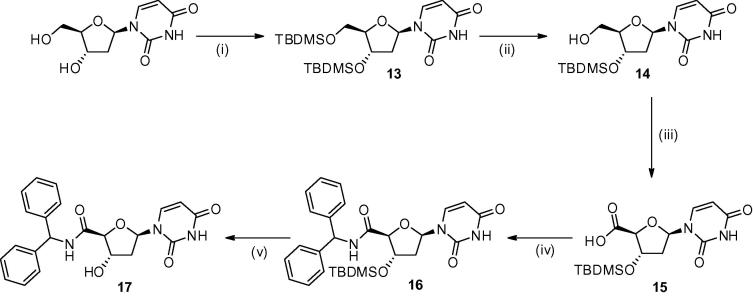
Synthetic route to the cyclic amide derivatives: (i) TBDMSCl, imidazole, THF/DMF, rt, 3 h, 72%-quantitative; (ii) PPTS, CH_3_OH, rt, 16 h, 30%; (iii) BAIB, TEMPO, CH_3_CN/H_2_O, rt, 14 h, 91%; (iv) (C_6_H_5_)_2_(CH)NH_2_, HOBt, TBTU, DIPEA, DMF, rt,16 h, 69%; (v) TBAF–Si, THF, rt, 24 h, 92%.

To prepare the cyclic amide analogues (trityl and diphenyl derivatives), 2′-deoxyuridine was protected at both the 5′ and the 3′-positions as *t*-butyldimethylsilyl (TBDMS) ethers (Scheme 3). This was followed by selective monodeprotection of the 5′ hydroxyl using pyridinium *para*-toluene sulphonate (PPTS). Oxidation of the alcohol was carried out in the presence of [bis(acetoxy)-iodo]benzene (BAIB) and 2,2,6,6-tetramethylpiperidine-1-oxyl (TEMPO) in water to give compound **15** in excellent yield.^15,16^ The final amidations were once again carried out with tritylamine and diphenylmethylamine. The diphenyl product (**16**) was obtained in 69% yield; however no triphenyl amide was obtained. It is likely that the trityl group was too bulky to be able to react at the crowded centre of the activated ester intermediate. It is anticipated that had the trityl amide been formed, then it is likely that this would have been more potent than that of the diphenyl analogue as has been seen previously, although larger and more lipophilic. The diphenyl derivative on its own however is sufficient to serve as an indicator as to whether these compounds would show any affinity for the *Pf*dUTPase. The final step was the removal of the protecting group from **16** in the presence of TBAF on silica.

## 2 Results and discussion

Biological activity was evaluated by testing all compounds against the recombinant *Pf*dUTPase and human dUTPase (*Hs*dUTPase) in order to determine inhibition constants and selectivity. Additionally compounds were screened in vitro against the chloroquine and pyrimethamine resistant, K1 strain of *Plasmodium falciparum* cultured in erythrocytes to evaluate antiplasmodial activity and the mammalian L6 cell line as a measure for cytotoxicity (Tables 3 and 4).

### 2.1 Acyclic analogues

The results for all the compounds tested are shown in Table 3 including the data for the straight chain acyclic analogues, **1f** and **1k**, for comparison.^9^ A number of conclusions can be drawn from this data:

• Compounds **3** and **4** have been included and show the requirement of the uracil in inhibition of *Pf*dUTPases (*K*_i_ = >100 and 1000 μM).
• The *N*-benzoyl derivative **6** (*K*_i_ >100 μM) is essentially inactive; removal of the benzoyl group from this trityl forward amide improves this value to 6.3 μM (compound **8**).
• The acid intermediate, **10**, which lacks the trityl or diphenyl moiety clearly shows low affinity for *Pf*dUTPase together with weak activity (25 μM) against *P*. *falciparum*.
• Comparison of ‘forward’ amides **7** and **8** with ‘reverse’ amides **11** and **12** shows that the ‘reverse’ amides are more potent against the *Pf*dUTPase (*K*_i_ = 11 and 6.3 μM vs *K*_i_ = 0.7 and 0.2 μM).
• The trityl ‘reverse amide’ (**12**) is also very comparable with the straight chain (unconstrained) trityl derivative, **1f** (*K*_i_ = 0.9 μM), whilst the diphenyl ‘reverse amide’ (**11**) is 8-fold more potent than the straight chain diphenyl compound, **1k** (*K*_i_ = 5.7 μM).

These results are noteworthy as a small change, such as reversing an amide bond has had a significant impact on the observed activities and illustrates the importance of correct orientation of any functionality. Additionally this is encouraging as it means that this extra rigidity in the chain is tolerated and the insertion of the amide group is not detrimental to activity if the correct orientation is attained.

The antiparasitic data remains constant for all the compounds ranging between 7 and 14 μM for **7**, **8**, **11** and **12**, although the selectivity decreases compared to mammalian cells. There was no correlation between inhibition of the enzyme and inhibition of the parasite growth.

### 2.2 Cyclic analogues

The results of cyclic amide **17** alongside the original trityl (**2b**) and diphenyl (**2c**) derivatives are shown in Table 4. Compound **17** showed some inhibition of the *Pf*dUTPase (*K*_i_ = 8.3 μM), and selectivity compared to the human dUTPase was retained (*K*_i_ = >100 μM). This may suggest that the diphenyl group is constrained in a sub-optimal conformation within the *Plasmodium* enzyme active site. Activity against the parasite is also poor (EC_50_ = >5 μM). In contrast to the acyclic series, this cyclic ‘reverse amide’ **17** was a weaker inhibitor of *Pf*dUTPase than the corresponding amine **2c**.

## 3 Conclusions

We have successfully prepared some conformationally restrained amide derivatives of the tritylated acyclic uridine derivatives. The aim of this work was to see if we could increase the potency of the compounds. Our ‘reverse amides’, **11** and **12**, showed similar or greater potency to the alkyl analogues against *Pfd*UTPase; whereas the ‘normal amides’ **7** and **8**, showed reduced activity. This suggests that the normal amides constrain the trityl/diphenyl group in a sub-optimal orientation and gives rise to unfavourable interactions in the active site.

The ‘reverse amides’ retain activity against the enzyme, but show reduced selectivity in the cellular assay, indicating off-target effects of these compounds.

## 4 Experimental section

### 4.1 Enzyme purification and inhibition assays

Both recombinant *P*. *falciparum* and human dUTPases were expressed in *E*. *coli* BL21 (DE3) cells which had been transformed with the pET11Pfdut and pET3Hudut (kindly provided by P.O. Nyman, Lund University, Sweden) expression vectors, respectively. For dUTPase purification, the same procedure was used for both the human and the *Plasmodium* enzymes. Cell pellets from a 2.8 L IPTG-induced culture were resuspended in 70 mL of buffer A (20 mM MES, 50 mM NaCl, 1 mM DTT, pH 5.5) containing a protease inhibitor cocktail. The cells were lysed by sonication, and the cell extract was cleared by centrifugation at 14,000 rpm for 30 min. The supernatant was loaded onto a 40 mL phosphocellulose (Whatman P-11) column at 4 °C and eluted with a 50 mM to 1 M NaCl gradient in buffer A. Protein was further purified by gel filtration chromatography on a Superdex 200 HA 10/30 column at 4 °C. Pooled fractions were concentrated by centrifugation at 4 °C and desalted using a PD-10 column. The enzyme was stored in 10 mM bicine and 5 mM MgCl_2,_ pH 8 at −80 °C. Purified fractions contained dUTPase of ⩾96% purity.

Nucleotide hydrolysis was monitored by mixing enzyme and substrate with a rapid kinetic accessory (Hi-Tech Scientific) attached to a spectrophotometer (Cary 50) and connected to a computer for data acquisition and storage as described previously.^17^ Protons, released through the hydrolysis of nucleotides, were neutralized by a pH indicator in weak buffered medium with similar p*K*_a_ and monitored spectrophotometrically at the absorbance peak of the basic form of the indicator. The ratio between the indicator and the buffer concentration was 50:2000 (M), and the absorbance changes were kept within 0.1 units. The indicator/buffer pair used was red cresol/bicine (pH 8, 573 nm). Assay mixes contained 30 nM *Pf*dUTPase, 50 μM dUTP, 5 mM MgCl_2_, 1 mg/mL BSA, and 100 mM KCl. *V*_max_ and *K*_Mapp_ were calculated by fitting the resulting data to the integrated Michaelis–Menten equation. The apparent *K*_M_ values were plotted against inhibitor concentration, and *K*_i_ values (Table 1) were obtained according to Eq. 1.

(1) $$K_{\text{Mapp}}=\frac{K_{\text{M}}}{K_{\text{i}}}[I]+K_{\text{M}}$$

Activity against *P*. *falciparum* K1 strain and cytotoxicity assessment against L6 cells (rat skeletal myoblast cells) was determined as previously reported.^9^

### 4.2 Chemistry

Solvents and reagents were purchased from commercial suppliers and used without further purification. Dry solvents were purchased in sure sealed bottles stored over molecular sieves. Reactions were performed in pre-dried apparatus under an atmosphere of argon unless otherwise stated. Normal phase TLC was carried out on pre-coated silica plates (Kieselgel 60 F_254_, BDH) with visualisation via either ninhydrin, PMA, or 254 nm UV light. Flash chromatography was performed using Combiflash Companion or Combiflash Rf and prepacked columns (silica gel) purchased from Redisep (Presearch), Silicycle (Anachem) or Grace Resolve. Preparative HPLC was performed using a Gilson (321-Pump, 153-UV–vis Detector) equipped with a Gilson liquid handler for injection and fraction collection and XBridge Prep C18, 5 μm, ODB, 19 × 100 mm column (Waters) with 0.1% ammonia in water (solvent A) and acetonitrile (solvent B) as mobile phase. Melting points (mp) were measured on a Gallenkamp melting point apparatus and are uncorrected. ^1^H NMR and ^13^C NMR spectra were recorded on a Bruker Avance DPX500 spectrometer or on a Bruker Avance DPX300 using the applied solvent simultaneously as internal standard. Deuterated solvents were purchased from Goss. Chemical shifts (*δ*) are given in ppm together with the multiplicity, relative frequency, coupling constants (*J*, Hz) and assignment. High resolution mass spectra were performed on a Bruker MicroTof mass spectrometer at University of Dundee. LC–MS analysis and chromatographic separation were conducted with a Bruker MicroTof mass spectrometer using an Agilent HPLC 1100 with a diode array detector in series. The column used was a Waters Xbridge C18, 3.5 μm particle size, 2.1 × 50 mm column and the compounds were eluted with a gradient of 5–95% Acetonitrile/H_2_O + 0.1% ammonia.

### 4.3 General procedure A for the synthesis of amides **3**–**4**, **11**–**12**

The relevant carboxylic acid (1 equiv), HOBt (1.4 equiv) and TBTU (1.4 equiv) were dissolved in DMF. To this was added DIPEA (3 equiv) and stirred at room temperature for 1 h. To the mixture, the amine (3.2 equiv) was added and the reaction left to stir at room temperature overnight under an atmosphere of Ar. The mixture was diluted with CHCl_3_ (25 mL) and extracted with H_2_O (5 × 30 mL). The organic layer was dried with MgSO_4_ and the solvent concentrated under reduced pressure. The product was purified by flash chromatography.

#### 4.3.1 General procedure B: Mitsunobu coupling of alcohols with 3-*N*-benzoyluracil for the synthesis of **5**–**6**

Polymer supported triphenylphosphine (2.5 equiv; 3 mmol/g) was swelled in THF for 15 min. To this was added the corresponding alcohol (1 equiv) and the *N*-3 benzoyluracil (2 equiv) which were shaken at room temperature for a further 15 min before DIAD (2 equiv) in THF was added to the mixture. The reaction was shaken until consumption of the alcohol was seen by TLC. The resin was then filtered off and washed with THF and the solvent removed under reduced pressure. The crude product was then purified by flash chromatography (Hexane/EtOAc 40:60 and/or CHCl_3_/CH_3_OH 95:5).

#### 4.3.2 General procedure C: hydrolysis of benzoyl esters for the synthesis of compounds **7**–**8**

The benzoyl ester intermediate was dissolved in a solution of 0.2 M sodium methoxide in CH_3_OH and the reaction stirred at room temperature overnight until the disappearance of the starting esters was observed (TLC). The solution was neutralised with Dowex H^+^ ion exchange resin, filtered and washed with methanol. The solution was concentrated in vacuo and the crude residue was purified by chromatography.

#### 4.3.3 *N*-(3-Hydroxypropyl)-2,2-diphenylacetamide (**3**)

Compound **3** was synthesised following general procedure A, using diphenyl acetic acid (1.00 g, 4.71 mmol), 3-amino-1-propanol (1.15 mL, 15.04 mmol), HOBt (1.07 g, 6.59 mmol), TBTU (2.12 g, 6.59 mmol) and DIPEA (2.46 mL, 14.13 mmol) dissolved in DMF (20 mL). The product was purified by flash chromatography (MeOH/CHCl_3_ 0 → 2%) to give the product as a white crystalline solid (508 mg, 40%). Purity by LCMS (UV chromatogram, 190–450 nm): >98%, Rt = 4.7 min; *R*_f_ = 0.10 (CHCl_3_/CH_3_OH 95:5); ^1^H NMR (500 MHz; CDCl_3_): *δ* 1.66 (m, 2H, HOCH_2_C*H*_2_), 3.10 (t, *J* = 6.3 Hz, 1H, OH), 3.46 (q, 6.2 Hz, 2H, NHC*H*_2_), 3.61 (q, *J* = 6.2 Hz, 2H, HOC*H*_2_), 4.96 (s, 1H, COC*H*), 6.06 (bs, 1H, NH), 7.27–7.38 (m, 10H, H–Ar); ^13^C NMR (125 MHz; CDCl_3_): *δ* 32.2 (CH_2_), 36.6 (CH_2_), 59.2 (CH_2_), 59.3 (CH), 127.4 (2 × CH–Ar), 128.9 (2 × CH–Ar), 139.2 (CH–Ar), 173.4 (CO); LRMS (ES^+^): *m*/*z* 270.1 [M+H]^+^, 539.3 [2M+H]^+^; HRMS (ES^+^): found 270.1483 [M+H]^+^ C_17_H_20_NO_2_^+^ requires 270.1489.

#### 4.3.4 *N*-(3-Hydroxypropyl)-2,2,2-triphenylacetamide (**4**)

Compound **4** was following using general procedure A, using triphenyl acetic acid (1.00 g, 3.47 mmol), 3-amino-1-propanol (845 μL, 11.1 mmol), HOBt (656.68 mg, 4.86 mmol), TBTU (1.56 g, 4.86 mmol) and DIPEA (1.82 mL, 10.41 mmol) dissolved in DMF (15 mL). Following extraction the product was sufficiently pure and did not require chromatography. **4** was obtained as an off white solid (1.03 g, 86%). Purity by LCMS (UV chromatogram, 190–450 nm): >98%, Rt = 5.1 min; *R*_f_ = 0.60 (CHCl_3_/CH_3_OH 90:10); ^1^H NMR (500 MHz; CDCl_3_): *δ* 1.66 (m, 2H, HOCH_2_C*H*_2_), 3.03 (t, *J* = 5.9 Hz, 1H, OH), 3.51 (q, *J* = 6.2 Hz, 2H, NHC*H*_2_), 3.56 (q, *J* = 5.6 Hz, 2H, HOC*H*_2_), 6.26 (bs, 1H, NH), 7.28–7.35 (m, 15H, *H*–Ar); ^13^C NMR (125 MHz; CDCl_3_): *δ* 32.3(CH_2_), 36.9 (CH_2_), 59.3 (CH_2_), 67.9 (CH), 127.1 (CH–Ar), 128.1–130.4 (2 × CH–Ar), 143.2 (CH–Ar), 174.7 (CO); LRMS (ES^+^): *m*/*z* 346.1 [M+H]^+^, 691.3 [2M+H]^+^; HRMS (ES^+^): found 346.1808 [M+H]^+^ C_23_H_24_NO_2_^+^ requires 346.1802.

#### 4.3.5 3-Benzoyl-(1-(3-diphenylacetylamino)propyl)uracil) (**5**)

Compound **5** was prepared following general procedure B from the alcohol **3** (499 mg; 1.85 mmol) and 3-*N*-benzoyl uracil (800 mg; 3.70 mmol) to give a white powder (0.317 g, 37%). *R*_f_ = 0.04 (EtOAc/Hexane 60:40); ^1^H NMR (500 MHz; CDCl_3_): *δ* 1.89 (m, 2H, NCH_2_C*H*_2_), 3.33 (q, *J* = 6.29 Hz, 2H, NHC*H*_2_), 3.73 (t, *J* = 6.32 Hz, 2H, NC*H*_2_), 4.90 (s, 1H, COC*H*), 5.80 (d, *J* = 7.96 Hz, 1H, NCHC*H*), 6.18 (t, *J* = 6.17 Hz, 1H, NH), 7.25–7.34 (m,10H, H–Ar), 7.36 (d, *J* = 7.97 Hz, 1H, NC*H*), 7.53 (t, *J* = 7.61 Hz, 2H, H–Ar), 7.69 (t, *J* = 7.46 Hz, 1H, H–Ar), 7.94 (d, *J* = 7.30 Hz, 2H, H–Ar); ^13^C NMR (125 MHz; CDCl_3_): *δ* 29.3 (CH_2_), 36.2 (CH_2_), 46.5 (CH_2_), 59.0 (CH), 102.4 (CH), 127.3–131.4 (C–Ar), 135.2 (C–Ar), 139.2 (C–Ar), 144.5 (CH), 150.2 (C), 162.3 (C), 168.8 (C), 172.6 (C);

LRMS (ES^+^): *m*/*z* 490.2 [M+Na]^+^, 957.4 [2M+Na]^+^; HRMS (ES^+^): found 468.1920 [M+H]^+^ C_28_H_26_N_3_O_4_^+^ requires 468.1918.

#### 4.3.6 3-Benzoyl-(1-(3-(triphenylacetylamino)propyl)uracil) (**6**)

Compound **6** was prepared following general procedure A from the alcohol **3** (200 mg; 0.579 mmol) and 3-*N*-benzoyl uracil **3** (253 mg; 1.16 mmol). This was obtained as a white powder (0.111 g, 35%). *R*_f_ = 0.16 (CHCl_3_/CH_3_OH 95:5); ^1^H NMR (500 MHz; (CD_3_)_2_SO): *δ* 1.75 (m, 2H, NCH_2_C*H*_2_), 3.17 (m, 2H, NHC*H*_2_), 3.60 (t, *J* = 6.9 Hz, 2H, NC*H*_2_), 5.84 (d, *J* = 8.0 Hz, 1H, NCHC*H*), 7.19–7.30 (m, 15H, H–Ar), 7.36 (t, *J* = 5. 9 Hz, 1H, NH), 7.58 (t, *J* = 7.9, 2H, H–Ar), 7.78 (m, 2H, NC*H*+H–Ar), 7.96 (d, *J* = 8.4 Hz, 2H, H–Ar); ^13^C NMR (125 MHz; (CD_3_)_2_SO): *δ* 28.9 (CH_2_), 37.2 (CH_2_), 46.7 (CH_2_), 67.7 (C), 101.1 (CH), 126.9–130.7 (C–Ar), 131.6 (C–Ar), 135.9 (CH), 144.2 (C–Ar), 147.1 (C–Ar), 150.0 (C), 162.7 (C), 170.1 (C), 172.7 (C); LRMS (ES^+^): *m*/*z* 544.2 [M+H]^+^; HRMS (ES^+^): found 544.2217 [M+H]^+^ C_34_H_30_N_3_O_4_^+^ requires 544.2231.

#### 4.3.7 1-(3-(Diphenylacetylamino)propyl)uracil (**7**)

Compound **7** was prepared following general procedure B from the 3-*N*-benzoyl protected intermediate **6** (298 mg; 0.638 mmol). The crude was purified by flash chromatography (CH_3_OH/CHCl_3_ 0 → 10%) to yield the product as a white solid (0.195 g, 84%). Purity by LCMS (UV chromatogram, 190–450 nm): >98%, Rt=3.1 min; *R*_f_ = 0.12 (CHCl_3_/CH_3_OH 95:5); Mp = 145–147 °C; ^1^H NMR (500 MHz; CDCl_3_): *δ* 1.87 (m, 2H, NCH_2_C*H*_2_), 3.31 (m, 2H, NC*H*_2_), 3.70 (t, *J* = 6.47 Hz, 2H, NC*H*_2_), 4.95 (s, 1H, COC*H*), 5.71 (dd, *J* = 7.9, 2.2 Hz, 1H, NCHC*H*), 6.29 (t, *J* = 6.11 Hz, 1H, NH), 7.24 (d, *J* = 7.9 Hz, NC*H*), 7.27–7.37 (m, 10H, H–Ar), 9.15 (s, 1H, CON*H*CO); ^13^C NMR (125 MHz; CDCl_3_): *δ* 29.3 (CH_2_), 36.1 (CH_2_), 46.0 (CH_2_), 59.1 (CH), 102.3 (CH), 127.3 (C–Ar), 128.8 (C–Ar), 139.3 (C–Ar), 144.6 (CH), 151.2 (C), 163.5 (C), 172.6 (C); LRMS (ES^+^): *m*/*z* 364.1 [M+H]^+^, 727.3 [2M+H]^+^; HRMS (ES^+^): found 364.1657 [M+H]^+^ C_21_H_22_N_3_O_3_^+^ requires 364.1656.

#### 4.3.8 1-(3-(Triphenylacetylamino)propyl)uracil (**8**)

Deprotection was carried out following general procedure C from the benzoyl intermediate **6** (73 mg; 0.166 mmol). The residue was purified by flash chromatography (CH_3_OH/CH_2_Cl_2_ 0 → 2%) to yield a white powder (0.029 g, 0.066 mmol, 40%). Purity by LCMS (UV chromatogram, 190–450 nm): 99%, Rt=3.5 min; *R*_f_ = 0.16 (CHCl_3_/CH_3_OH 95:5); mp: 154–156 °C; ^1^H NMR (500 MHz; (CD_3_)_2_SO): *δ* 1.67 (m, 2H, NCH_2_C*H*_2_), 3.13 (m, 2H, NC*H*_2_), 3.49 (t, *J* = 7.0 Hz, 2H, NC*H*_2_), 5.53 (d, *J* = 7.8 Hz, 1H, NCHC*H*), 7.21–7.33 (m, 15H, H–Ar), 7.50 (d, *J* = 7.8 Hz, 1H, NCH), 11.24 (s, 1H,CON*H*CO); ^13^C NMR (125 MHz; (CD_3_)_2_SO): *δ* 29.0 (CH_2_), 37.1 (CH_2_), 45.9 (CH_2_), 67.8 (CH), 101.3 (CH), 126.9 (C–Ar), 128.2 (C–Ar), 130.6 (C–Ar), 144.2 (C–Ar), 146.1 (CH), 151.4 (C), 164.1 (C), 172.7 (C); LRMS (ES^+^): *m*/*z* 440.2 [M+Na]^+^, *m*/*z* 901.4 [2M+Na]^+^; HRMS (ES^+^): found 440.1950 [M+H]^+^ C_27_H_26_N_3_O_3_^+^ requires 440.1969.

#### 4.3.9 1-(Carboxypropyl)uracil (**10**)^18^

To a mixture of compound **9** (2.92 g, 8.84 mmol) suspended in THF (25 mL) was added NaOH (90 mL. 88.40 mmol, 1 M) and the mixture stirred vigorously at rt overnight. The solvents were removed under reduced pressure and the residue taken up in H_2_O (50 mL) and acidified with HCl (1 M), followed by extraction with CHCl_3_ (3 × 100 mL). The aqueous layer was concentrated and the residue purified by flash chromatography (0 → 20% CH_3_OH/CHCl_3_ + 0.3% TFA) to give a white solid (1.13 g, 5.71 mmol, 65%). Purity by LCMS (UV chromatogram, 190–450 nm): >98%, Rt = 0.6 min; *R*_f_ = 0.15 (CHCl_3_/ CH_3_OH 80:20); Mp: 174–176 °C; ^1^H NMR (500 MHz; CDCl_3_): *δ* 1.80 (m, 2H, NCH_2_C*H*_2_), 2.24 (t, *J* = 7.4 Hz, 2H, NC*H*_2_), 3.68 (t, *J* = 7.0 Hz, 1H, COC*H*_2_), 5.54 (dd, *J* = 2.3, 7.8 Hz, 1H, NCHC*H*), 7.62 (d, *J* = 7.8 Hz, 1H, NC*H*), 11.23 (bs, 1H, NH), 12.17 (bs, OH); ^13^C NMR (125 MHz; CDCl_3_): *δ* 24.3 (CH_2_), 31.0 (CH_2_), 47.4 (CH_2_), 101.4 (CH), 146.1 (CH), 151.4 (C), 164.2 (C), 174.2 (C); LRMS (ES^+^): *m*/*z* 199.0 [M+H]^+^, 397.1 [2M+H]^+^; HRMS (ES^+^): found 199.0718 [M+H]^+^ C_8_H_11_N_2_O_4_^+^ requires 199.0713.

#### 4.3.10 1-[3-(Benzhydrylaminocarbonyl)propyl]uracil (**11**)

Compound **11** was synthesised using following procedure A, using acid **10** (400 mg, 2.02 mmol), benzhydryl amine (1.20 mL, 6.46 mmol), HOBt (384 mg, 2.83 mmol), TBTU (9.09 mg, 2.83 mmol) and DIPEA (1.06 mL, 6.06 mmol) dissolved in DMF (20 mL). The product was purified by flash chromatography (0 → 5% CH_3_OH/CH_2_Cl_2_ + 3% Et_3_N) as an off-white solid (102 mg; 0.281 mmol, 14%). Purity by LCMS (UV chromatogram, 190–450 nm): 97%, Rt = 4.2 min; *R*_f_ = 0.23 (CHCl_3_/ CH_3_OH 95:5); ^1^H NMR (500 MHz; CDCl_3_): *δ* 2.06 (m, Hz, 2H, NCH_2_C*H*_2_), 2.35 (t, *J* = 6.8 Hz, 2H, NC*H*_2_), 3.82 (t, *J* = 6.8 Hz, 2H, COC*H*_2_), 5.66 (d, *J* = 7.9 Hz, 1H, NCHC*H*), 6.26 (d, *J* = 8.1 Hz, 1H, NHC*H*), 6.57 (d, *J* = 8.1 Hz, 1H, CHN*H*CO); 7.21 (d, *J* = 7.9 Hz, 1H, NC*H*), 7.25–7.37 (m, 10H, H–Ar), 8.65 (bs, 1H, CON*H*CO); ^13^C NMR (125 MHz; CDCl_3_): *δ* 24.8 (CH_2_), 32.4 (CH_2_), 47.8 (CH_2_), 57.1 (CH), 102.4 (CH), 127.3–128.7 (C–Ar × 3), 141.4 (C–Ar), 144.7 (CH), 151.1 (CO), 163.3 (CO), 170.6 (CO); LRMS (ES^+^): *m*/*z* 364.1 [M+H]^+^, 749.3 [2M+Na]^+^; HRMS (ES^+^): found 364.1638 [M+H]^+^ C_21_H_22_N_3_O_3_^+^ requires 364.1656.

#### 4.3.11 1-[3-(Tritylaminocarbonyl)propyl]uracil (**12**)

Compound **12** was synthesised following general procedure A, using acid **10** (482 mg, 2.43 mmol), tritylamine (2.02 g, 7.78 mmol), HOBt (459 mg, 3.40 mmol), TBTU (1.09 g, 3.40 mmol) and DIPEA (1.30 mL, 7.29 mmol) dissolved in DMF (20 mL). The product was purified by flash chromatography (0 → 10% CH_3_OH/CH_2_Cl_2_ + 3% Et_3_N, then 0–100% EtOAc/Hexane) as a white solid (179 mg, 0.408 mmol, 18%). Purity by LCMS (UV chromatogram, 190–450 nm): >98%, Rt = 3.5 min; *R*_f_ = 0.36 (CHCl_3_/CH_3_OH 95:5); ^1^H NMR (500 MHz; CDCl_3_): *δ* 2.00 (m, 2H, NCH_2_C*H*_2_); 2.37 (t, *J* = 6.6 Hz, 2H, COC*H*_2_), 3.72 (t, *J* = 6.9 Hz, 2H, NC*H*_2_), 5.64 (d, *J* = 7.9 Hz, 1H, NCHC*H*), 6.75 (s, 1H, CHN*H*), 7.12 (d, *J* = 7.9 Hz, 1H, NC*H*), 7.21–7.34 (m, 15H, H–Ar), 8.49 (bs, 1H, CON*H*CO); ^13^C NMR (125 MHz; CDCl_3_): *δ* 13.9 (CH_2_), 24.6 (CH_2_), 33.1 (CH_2_), 64.8 (CH), 102.1 (CH), 127.2–130.0 (C–Ar × 3), 144.4 (CH), 145.0 (CO), 170.6 (CO), 176.2 (CO); LRMS (ES^+^): *m*/*z* 243.0 [Ph_3_C]^+^, 440.1 [M+H]^+^, 896.4 [M+NH_4_]^+^; HRMS (ES^+^): found 440.1977 [M+H]^+^ C_27_H_26_N_3_O_3_^+^ requires 440.1969.

#### 4.3.12 3′,5′-O-Di(*tert*-butyldimethylsilyl)-2′-deoxyuridine (**13**)^19^

To a solution of 2′-deoxyuridine (1.00 g, 4.39 mmol) in THF/DMF (1:1) (10 mL) was added imidazole (1.20 g, 17.56 mmol) followed by TBDMSCl (1.46 g, 9.66 mmol), and stirred at rt for 3 h. The mixture was diluted with EtOAc (25 mL) and washed with H_2_O (3 × 25 mL). The organic layer was dried (MgSO_4_), concentrated and purified by flash chromatography (CH_3_OH/CH_3_Cl 0 → 20%) to yield the product as a white crystalline foam (1.44 g, 3.16 mmol, 72%). Purity by LCMS (UV chromatogram, 190–450 nm): >98%, Rt = 5.8 min; *R*_f_ = 0.51 (CH_3_OH/CHCl_3_ 10:90); Mp: 45–47 °C; ^1^H NMR (500 MHz, CDCl_3_): *δ* 0.00 (d, *J* = 2.8 Hz, 6H, Si(C*H*_3_)_2_), 0.03 (s, 6H, Si(C*H*_3_)_2_), 0.79 (s, 9H, (C*H*_3_)_3_), 0.81 (s, 9H, (C*H*_3_)_3_), 1.96 (m, 1H, C*H*H-2′), 2.22 (m, 1H, CH*H*-2′), 3.66 (dd, *J* = 11.2, 1.8 Hz 1H, C*H*H-5′), 3.81 (dd, *J* = 8.1, 2.2 Hz, 1H, CH*H*-5′ + H-4′), 4.31 (m, 1H, H-3′), 5.58 (d, *J* = 8.2 Hz, 1H, H-5), 6.19 (t, *J* = 6.2 Hz, 1H, H-1′), 7.80 (d, *J* = 8.2 Hz, 1H, H-6), 8.31 (bs, 1H, NH-3); ^13^C NMR (125 MHz, CDCl_3_): *δ* [−5.5]–[−4.6] (Si((CH_3_)_2_) × 2), 18.0–18.3 ((CH_3_)_3_ × 2), 25.7–25.8 (SiC × 2), 41.8(CH_2_-2′), 62.4 (CH-3′), 71.1 (CH_2_-5′), 85.1 (CH-4′), 87.7 (CH-1′), 102.1 (CH-5), 140.2 (CH-6), 150.0 (C-2), 163.0 (C-4); LRMS (ES^+^): *m*/*z* 457.2 [M+H]^+^, 479.3 [M+Na]^+^, 913.5 [2M+H]^+^; HRMS (ES^+^): found 457.2538 [M+H]^+^ C_21_H_41_N_2_O_5_Si_2_^+^ requires 457.2549.

#### 4.3.13 3′-O-(*tert*-butyldimethylsilyl)-2′-deoxyuridine (**14**)

**13** (1.43 g, 3.14 mmol) and PPTS (4.21 g, 16.75 mmol) were combined in CH_3_OH (30 mL) and stirred at rt until reaction completion. This was concentrated, the residue suspended in EtOAc (30 mL) and washed with H_2_O (7 × 30 mL). The organic layer was dried (MgSO_4_), concentrated to give the product (0.322 g, 0.942 mmol, 30%) which did not require further purification. *R*_f_ = 0.44 (CH_3_OH/CHCl_3_ 10:90); ^1^H NMR (500 MHz, CDCl_3_): *δ* 0.00 (s, 6H, Si(C*H*_3_)_2_), 0.80 (s, 9H, SiC(C*H*_3_)_3_), 2.20 (m, 2H, H-2′), 3.68 (m, 1H, C*H*H-5′), 3.85 (m, 2H, CH*H*-5′ + H-4′), 4.40 (m, 1H, H-3′), 5.65 (dd, *J* = 8.1, 1.8 Hz 1H, H-5), 6.08 (t, *J* = 6.6 Hz, 1H, H-1′), 7.55 (d, *J* = 8.1 Hz, 1H, H-6), 8.60 (bs, 1H, N*H*-3); ^13^C NMR (125 MHz, CDCl_3_): *δ* −4.8 (Si(CH_3_)_2_)), 17.9 (SiC), 25.7 ((C*H*_3_)_3_), 40.8(CH_2_-2′), 61.9 (CH_2_-5′), 71.4 (CH-3′), 86.8 (CH-1′), 87.5 (CH-4′), 102.5 (CH-5), 141.1 (CH-6), 150.1 (C-2), 163.0 (C-4); LRMS (ES^+^): *m*/*z* 343.1 [M+H]^+^, 685.2 [2M+H]^+^.

#### 4.3.14 3′-O-(*tert*-butyldimethylsilyl)-2′-deoxyuridine-5′-carboxylic acid (**15**)

TEMPO (0.031 g, 0.198 mmol) and BAIB (0.728 g, 2.26 mmol) were added to a solution of **14** (0.322 g, 0.942 mmol) in CH_3_CN/H_2_O (1:1) (20 mL). This was left to stir overnight at rt. The mixture was diluted with EtOAc (15 mL) and washed with H_2_O (3 × 10 mL). The organic layer was dried (MgSO_4_), concentrated and purified by flash chromatography (EtOAc/Hexane 0 → 100%) to give **15** (0.082 g, 0.230 mmol, 24%) as a white powder. Purity by LCMS (UV chromatogram, 190–450 nm): >98%, Rt = 0.5 min; *R*_f_ = 0.13 (CH_3_OH/CHCl_3_ 10:90); ^1^H NMR (500 MHz, CDCl_3_): *δ* 0.01 (d, *J* = 6.5 Hz, 6H, Si(C*H*_3_)_2_), 0.77 (s, 9H, SiC(C*H*_3_)_3_), 1.97 (m, 1H, C*H*H-2′), 2.20 (m, 1H, CH*H*-2′), 4.37 (s, 1H, H-3′), 4.51 (d, *J* = 4.3 Hz, 1H, H-4′), 5.72 (d, *J* = 8.0 Hz, 1H, H-5), 6.20 (dd, *J* = 9.1, 5.1 Hz, 1H, H-1′), 7.98 (d, *J* = 8.1 Hz, 1H, H-6), 8.69 (bs, 1H, N*H*-3); ^13^C NMR (125 MHz, CDCl_3_): *δ* −4.9 Si(CH_3_)_2_), 18.0 (SiC), 25.6 ((C*H*_3_)_3_), 39.5(CH_2_-2′), 75.8 (CH-3′), 85.4 (CH-1′), 88.9 (CH-4′), 102.7 (CH-5), 141.8 (CH-6), 150.2 (C-2), 163.5 (C-4), 172.9 (C-5′); LRMS (ES^+^): *m*/*z* 357.1 [M+H]^+^, 713.3 [2M+H]^+^; LRMS (ES^−^): *m*/*z* 355.1 [M−H]^+^; HRMS (ES^+^): found 357.1482 [M+H]^+^ C_15_H_25_N_2_O_6_Si^+^ requires 357.1476.

#### 4.3.15 3′-O-(*tert*-butyldimethylsilyl)-2′-deoxyuridine-5′-*N*-benzhydryl carboxamide (**16**)

Compound **16** was synthesised following general procedure A, using acid **15** (0.040 g, 0.124 mmol), benzhydrylamine (0.073 g, 0.397 mmol), HOBt (0.026 g, 0.149 mmol), TBTU (0.048 g, 0.149 mmol) and DIPEA (0.048 g, 0.372 mmol) dissolved in DMF (10 mL). Upon completion, the mixture was diluted with CH_2_Cl_2_ (15 mL) and washed with H_2_O (3 × 15 mL). The organic portion was dried (MgSO_4_) and concentrated. Purification by HPLC (Rt = 10.9 min, 1 min hold 95% A, 3.5 min ramp to 95% B, 3.5 min hold 95% B) gave **16** (0.045 g, 0.086 mmol, 69%) as a white powder. *R*_f_ = 0.32 (CH_3_OH/CHCl_3_ 10:90); Mp: 94–96 °C; ^1^H NMR (500 MHz, CDCl_3_): *δ* 0.00 (d, *J* = 5.0 Hz, 6H, Si((C*H*_3_)_2_), 0.78 (s, 9H, SiC(C*H*_3_)_3_), 2.02 (dd, *J* = 12.1, 4.9 Hz, 1H, C*H*H-2′), 2.22 (m, 1H, CH*H*-2′), 4.27 (s, 1H, H-4′), 4.58 (d, *J* = 4.7 Hz, 1H, H-3′), 5.42 (d, *J* = 8.1 Hz, 1H, H-5), 6.14–6.19 (m, 2H, H-1′ + C*H*NH), 7.13–7.25 (m, 12H, H–Ar + H-6 + N*H*-3); ^13^C NMR (125 MHz, CDCl_3_): *δ* −4.8 ((CH_3_)_2_), 18.0 (SiC), 25.7 ((CH_3_)_3_), 38.5(C-2′), 56.8 (C*H*_3_)_2_), 75.1 (CH-3′), 87.1 (CH-4′), 88.6 (CH-1′), 103.2 (CH-5), 127.1–128.9 (CH–Ar × 3), 140.6–141.3 (CH-6 + C-Ar), 150.4 (C-2), 163.1 (C-4), 168.8 (C-5′); LRMS (ES^+^): *m*/*z* 167.0 [Ph_2_C]^+^, 522.2 [M+H]^+^; HRMS (ES^+^): found 522.2405 [M+H]^+^ C_28_H_36_N_3_O_5_Si^+^ requires 522.2405.

#### 4.3.16 2′,5′-Dideoxyuridine-5′-*N*-benzhydryl carboxamide (**17**)

Silyl protected compound, **16** (0.351 g, 0.674 mmol) was dissolved in THF (20 mL). To this was added TBAF on silica (1.13 g, 1.70 mmol, 1.5 mmol F^−^/g) and stirred at rt for 24 h. A further portion of TBAF on silica was added and stirred for a further 7 h. The mixture was filtered and washed 5% CH_3_OH/CHCl_3_ and the filtrate dried (MgSO_4_), concentrated and purified by flash chromatography (CH_3_OH/CHCl_3_ 0 → 5%) to give **17** (0.253 g, 92%) as a white powder. Purity by LCMS (UV chromatogram, 190–450 nm): 96%, Rt = 4.3 min; *R*_f_ = 0.29 (CH_3_OH/CHCl_3_ 10:90); ^1^H NMR (500 MHz, (CD_3_)_2_SO): *δ* 2.15–2.17 (m, 2H, H-2′), 4.32 (s, 1H, H-3′) 4.49 (s, 1H, H-4′), 4.63–5.65 (dd, *J* = 8.1, 1.8 Hz, 1H, H-5), 5.66–5.67 (d, *J* = 4.4 Hz, 1H, OH-3′), 6.14–6.16 (d, *J* = 8.5 Hz, 1H, C*H*NH), 6.32–6.35 (t, *J* = 7.1 Hz, 1H, H-1′), 7.26–7.37 (m, 10H, H–Ar), 8.34–8.35 (d, *J* = 8.1 Hz, 1H, H-6), 9.27–9.29 (d, *J* = 8.5 Hz, 1H, CHN*H*), 11.31 (s, 1H, NH-3); ^13^C NMR (125 MHz, (CD_3_)_2_SO): *δ* 38.9 (CH_2_-2′), 56.0(CHNH), 73.7 (3′-CH), 85.1 (4′-CH), 85.6 (CH-1′), 101.9 (CH-5), 127.1 (CH–Ar), 127.2 (CH–Ar), 128.4 (CH–Ar), 141.1 (CH-6), 128.4 (C–Ar), 150.5 (C-2), 163.0 (C-4), 169.8 (C-5′); LRMS (ES^+^): *m*/*z* 167.0 [Ph_2_C]^+^, 408.2 [M+H]^+^; 815.3 [2M+H]^+^; HRMS (ES^+^): found 408.1538 [M+H]^+^ C_22_H_22_N_3_O_5_^+^ requires 408.1554.

## Figures and Tables

**Table 1:** Biological results for selected acyclic PfdUTPase inhibitors as previously reported^9^

**Figure:**
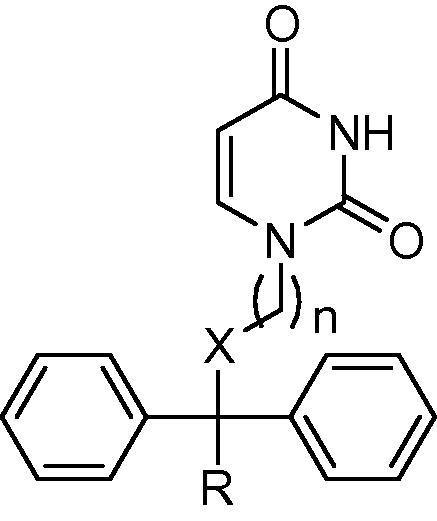

Compound	X	*n*	R	MW	*c *log* P*	Enzyme assay *K*_i_ (μM)			In vitro assays EC_50_ (μM)		
						*Pf*dUTPase	*Hs*dUTPase	SI^a^	*P.f*.^b^	L6 cells^c^	SI^d^
**1a**	O	3	Ph	412	3.7	87	313	4	7.5	34	5
**1b**	O	4	Ph	426	4.1	1.6	>1000	>617	4.9	42	7
**1c**	O	5	Ph	440	4.7	2.0	>1000	>1	1.1	24	21
**1d**	O	6	Ph	454	5.2	2.3	476	50	2.3	44	19
**1e**	NH	3	Ph	411	3.5	0.2	1.4	7	4.4	107	24
**1f**	NH	4	Ph	425	3.8	0.9	>1000	>1111	3.8	33	9
**1g**	NH	5	Ph	439	4.3	4.3	>1000	>233	2.2	39	18
**1h**	NH	6	Ph	453	4.9	1.8	>1000	>556	1.1	40	36
**1i**	NH	3	H	335	2.2	5.7	10	2	5.5	269	49
**1j**	O	4	H	350	2.9	0.5	49	98	14	104	7
**1k**	NH	4	H	349	2.6	5.7	63	11	2.6	99	38

a Selectivity index (SI) for enzyme calculated as [*K*_i_*Hs*dUTPase/*K*_i_*Pf*dUTPase].

b *P*. *falciparum* K1, a chloroquine and pyrimethamine resistant strain.

c Cytotoxicity on rat L6 myoblasts.

d Cell selectivity index (SI) was calculated as [EC_50_ L6/EC_50_*P*. *falciparum*]. Controls: for *P*. *falciparum,* chloroquine, EC_50_ = 0.1 μM; for cytotoxicity, podophyllotoxin, EC_50_ = 0.012 μM. The EC_50_ values are the means of two independent assays; the individual values vary less than a factor 2.

**Table 2:** Biological results for selected cyclic PfdUTPase inhibitors as previously reported^12,13^

**Figure:**
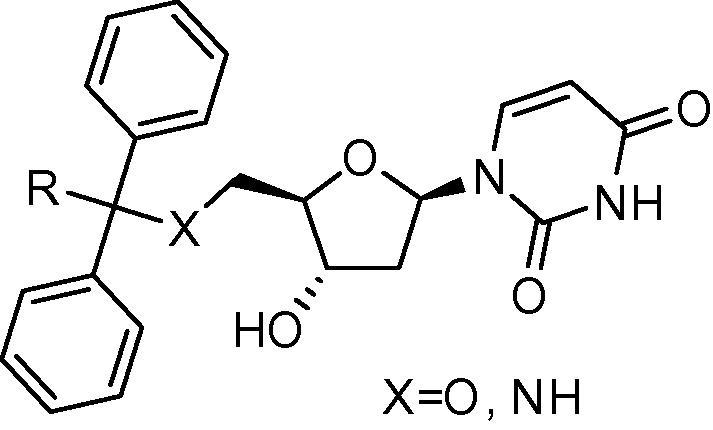

Compd	X	R	MW	*c *log* P*	Enzyme assay *K*_i_ (μM)			In vitro assays EC_50_ (μM)		
					*Pf*dUTPase	*Hs*dUTPase	SI^a^	*P.f*.^b^	L6 cells^c^	SI^d^
**2a**	O	Ph	470	3.0	1.8	18	10	6.0	192	32
**2b**	NH	Ph	469	2.8	0.2	46	232	4.5	—	—
**2c**	NH	H	393	1.6	0.2	>100	>500	12	>229	>20

a Selectivity index (SI) for enzyme calculated as [*K*_i_*Hs*dUTPase/*K*_i_*Pf*dUTPase].

b *P*. *falciparum* K1 chloroquine and pyrimethamine resistant strain.

c Cytotoxicity on rat L6 myoblasts.

d Cell selectivity index (SI) was calculated as [EC_50_ L6/EC_50_*P*. *falciparum*]. Controls: for *P*. *falciparum,* chloroquine, EC_50_ = 0.1 μM; for cytotoxicity, podophyllotoxin, EC_50_ = 0.012 μM. The EC_50_ values are the means of two independent assays; the individual values vary less than a factor 2.

**Table 3:** Activity data for the acyclic derivatives

Compound No.	Structure	Enzyme assay, *K*_i_ (μM)			In vitro assays, EC_50_ (μM)		
		*P.fal*	*H. sap.*	SI^a^	*P.fal.*^b^	L6 cells^c^	SI^d^
**3**		>100	1000	13	19	18	1.0
**4**		1000	1000	1	9.8	19	1.9
**6**		>100	1000	56	6.7	9	1.4
**7**		11	286	26	14	18	1.3
**8**		6.3	1000	159	11	18	1.6
**10**		>100	1000	400	25	18	0.7
**11**		0.7	1000	1429	13	16	1.2
**12**		0.2	1000	4545	7.2	4	0.6
**1f**^e^		0.9	>1000	>1111	3.8	33	9
**1k**^e^		5.7	63	11	2.6	99	38

a Selectivity index (SI) for enzyme calculated as [*K*_i_*Hs*dUTPase/*K*_i_*Pf*dUTPase].

b *P*. *falciparum* K1 chloroquine and pyrimethamine resistant strain.

c Cytotoxicity on rat L6 myoblasts.

d Cell selectivity index (SI) was calculated as [EC_50_ L6/EC_50_*P*. *falciparum*]

e Compounds **1f** and **1k** have previously been published,^9^ however have been included for comparative purposes. Controls: for *P*. *falciparum,* chloroquine, EC_50_ = 0.1 μM; for cytotoxicity, podophyllotoxin, EC_50_ = 0.012 μM. The EC_50_ values are the means of two independent assays, the individual values vary less than a factor 2.

**Table 4:** Biological results for selected cyclic and acyclic PfdUTPase inhibitors

Compound No.	Structure	*c *log* P*	Enzyme assay, *K*_i_ (μM)			In vitro assays, EC_50_ (μM)		
			*P.fal.*	*H. sap.*	SI^a^	*P.fal.*^b^	Tox^c^	SI^d^
**2b**^e^		2.8	0.2	46	230	4.5	—	—
**2c**^e^		1.6	0.2	>100	>500	12	>229	>20
**17**		1.4	8.3	>100	>12	>5	>90	—


a Selectivity index (SI) for enzyme calculated as [*K*_i_*Hs*dUTPase/*K*_i_*Pf*dUTPase].

b *P*. *falciparum* K1 chloroquine and pyrimethamine resistant strain.

c Cytotoxicity on rat L6 myoblasts.

d Cell selectivity index (SI) was calculated as [EC_50_ L6/EC_50_*P*. *falciparum*].

e Compounds **2b** and **2c** have previously been published,^12,13^ however have been included for comparative purposes. Controls: for *P*. *falciparum,* chloroquine, EC_50_ = 0.1 μM; for cytotoxicity, podophyllotoxin, EC_50_ = 0.012 μM. The EC_50_ values are the means of two independent assays; the individual values vary less than a factor 2.
